# Antidiabetic and Liver Histological and Ultrastructural Effects of *Cynara scolymus* Leaf and Flower Head Hydroethanolic Extracts in Nicotinamide/Streptozotocin-Induced Diabetic Rats

**DOI:** 10.1155/2023/4223026

**Published:** 2023-04-25

**Authors:** Osama M. Ahmed, Asmaa A. Abdel Fattah, Manal Abdul-Hamid, Ayman M. Abdel-Aziz, Hader I. Sakr, Ahmed A. Damanhory, Samraa H. Abdel-Kawi, Nehmat Ghaboura, Moaaz M. Y. Awad

**Affiliations:** ^1^Physiology Division, Department of Zoology, Faculty of Science, Beni-Suef University, P.O. 62521, Beni-Suef, Egypt; ^2^Cell Biology, Histology and Genetics Division, Department of Zoology, Faculty of Science, Beni-Suef University, P.O. 62521, Beni-Suef, Egypt; ^3^Zoology Department, Faculty of Science, Fayoum University, Fayoum 63514, Egypt; ^4^Department of Medical Physiology, Faculty of Medicine, Cairo University, Cairo, Egypt; ^5^Department of Medical Physiology, Medicine Program, Batterjee Medical College, Jeddah 21442, Saudi Arabia; ^6^Department of Biochemistry, Faculty of Medicine, Al-Azhar University, Cairo, Egypt; ^7^Department of Biochemistry, Medicine Program, Batterjee Medical College, Jeddah 21442, Saudi Arabia; ^8^Department of Medical Histology and Cell Biology, Faculty of Medicine, Beni-Suef University, Beni-Suef, Egypt; ^9^Department of Pharmacy Practice, Pharmacy Program, Batterjee Medical College, P.O. Box 6231, Jeddah 21442, Saudi Arabia; ^10^Department of Anatomy, Medicine Program, Batterjee Medical College, Jeddah, Saudi Arabia; ^11^Department of Anatomy, Damietta Faculty of Medicine, Al-Azhar University, Damietta, Egypt

## Abstract

This study aims to investigate the effect of hydroethanolic extracts of *Cynara scolymus (C. scolymus)* leaf (CLHE) and *C. scolymus* flower (CFHE) on the hepatic histopathological lesions and functional biochemical changes induced by type 2 diabetes mellitus (T2DM). The rat model of T2DM was induced by intraperitoneal injection of streptozotocin (STZ) in a dose of 60 mg/kg for 15 minutes following nicotinamide (NA) (60 mg/kg). The rats were allocated into four groups: group 1 (negative control), group 2 (diabetic control), group 3 (diabetic rats supplemented with 100 mg/kg/day CLHE), and group 4 (diabetic rats supplemented with 100 mg/kg/day CFHE). Treatment with CLHE and CFHE, for the study duration of 28 days, significantly improved the deteriorated hepatic glycogen content, glycogen phosphorylase, glucose-6-phosphatase activities, serum fructosamine levels, lipid profile, aspartate transaminase activities, and alanine transaminase activities as well as serum insulin and C-peptide levels. The elevated liver lipid peroxidation and the decreased activities of superoxide dismutase and glutathione peroxidase were significantly alleviated. The elevated expression of the proinflammatory cytokine tumor necrosis factor-*α* in the liver of diabetic rats was significantly reduced by treatments with CLHE and CFHE. NA/STZ-induced T2DM exhibited hepatic histopathological changes in the form of disordered hepatocytes, cytoplasm dissolution, and mononuclear leukocytic infiltration. The electron microscopic ultrastructure study revealed damaged mitochondria with ill-defined cristae and fragmentation of the rough endoplasmic reticulum. Treatments with CLHE and CFHE remarkably amended these histopathological and EM ultrastructural changes. In conclusion, both CLHE and CFHE may have antidiabetic and improvement effects on the liver function and structural integrity, which may be mediated, at least in part, *via* suppression of inflammation and oxidative stress and enhancement of the antioxidant defence system.

## 1. Introduction

Diabetes mellitus (DM) is a chronic major disorder of carbohydrate metabolism due to impaired insulin secretion and/or insulin resistance; however, lipid and protein metabolisms are also defective [1–3]. It is a complex metabolic disease with increasing incidence in the adult population; the number is predicted to increase to 643 million by 2030 and 783 million by 2045 [4]. Thereby, it is one of the principal public health problems of the 21^st^ century that threatens populations worldwide. It was classified into four types, with type 2 DM (T2DM) being the most common, accounting for approximately 95% of all types of DM [5, 6]. Possible mechanisms of metabolic stress-induced *β*-cell apoptosis in T2DM include endoplasmic reticulum (ER) stress [7], oxidative stress [2, 8], and ceramide production [9, 10], and these culminate in the stimulation of the intrinsic or mitochondrial apoptotic pathway [11, 12].

Dyslipidemia consistent with insulin resistance (IR) and T2DM is featured by elevated triacylglycerols (TAGs) and decreased high-density lipoprotein-cholesterol (HDL-C) levels [2], which are, in addition to low-density lipoprotein-cholesterol (LDL-C) high levels, risk factors for coronary heart disease [13]. Free fatty acids (FFAs), diacylglycerol (DAGs), and TAGs that accumulate intracellularly are the lipids responsible for the pathophysiology of IR [14, 15]. Individuals with T2DM have a high incidence of liver function test abnormalities, with 23–57% prevalence [16]. Serum or plasma aminotransferases, *viz* aspartate transaminase (AST) and alanine transaminase (ALT), measure the intracellular hepatic enzymes leaked into the blood and serve as markers for liver injury [17].

Plant extracts and constituents with antidiabetic potentials are yet to be formulated commercially as modern medicines, although they have been attracting public approval and praise for their therapeutic activities in traditional medicines [2, 3, 15]. Artichoke or *Cynara scolymus* (*C. scolymus*) L., belonging to the family Asteraceae, is an ancient medicinal plant whose therapeutic potential was well-identified by the Romans, ancient Egyptians, and Greeks [18]. The genus *Cynara* (*C*.) originates from the Mediterranean regions and includes 2 varieties, globe artichoke (*C. cardunculus* var. *Scolymus* L.) and cardoon (*C. cardunculus* var. *altilis* DC), as well as their ancestors, wild cardoon (*C. cardunculus* L. var. *sylvestris* (Lamk) Fiori) [19]. Our previous publication reported that artichoke leaf and flower extract have antihyperglycemic actions in type 2 diabetic rats [20]. Artichoke leaf extracts have traditionally been used to treat dyspeptic symptoms, increase bile flow, and exert hepatoprotective, lipid-lowering, antioxidant, and antispasmodic effects [21].

In conductance with the previous literature, the present study was designed to assess the effects of *C. scolymus* leaf hydroethanolic extract (CLHE) and *C. scolymus* flower hydroethanolic extract (CFHE) on glycemic state, lipid profile, liver function, and histological integrity in nicotinamide (NA)/streptozotocin (STZ)-induced diabetic Wistar rats.

## 2. Materials and Methods

### 2.1. Chemicals

NA and STZ were obtained from Sigma Chemical Company (USA). Proteinase K was obtained from Bioline USA Inc., Taunton, MA, USA. All other chemicals used in this investigation were ultrapure and of an analytical grade.

### 2.2. Plant Extraction

Artichoke or *Cynara scolymus* L. was collected from Beheira (Egypt), washed, and air-dried at room temperature for three weeks. The plant was authenticated by Dr. Mohamed A. Abdallah Fadl, Associate Professor of Taxonomy, Botany Department, Faculty of Science, Beni-Suef University, Egypt. Plant samples were deposited in the Herbarium of Botany Department, Faculty of Science, Beni-Suef University, Egypt. By using, the National Center for Biotechnology Information (NCBI) taxonomy database, Taxonomy ID: 59895 (NCBI: txid59895) (https://www.ncbi.nlm.nih.gov/Taxonomy/Browser/wwwtax.cgi?id=59895), an electrical grinder later powdered the dried leaves and flower heads. The powder was soaked in 70% ethyl alcohol separately for 72 hours at room temperature. The mixtures were filtered *via* Whatman's filter papers and evaporated under reduced pressure using a rotatory evaporator to obtain crude extracts. The hydroethanolic extracts (CLHE and CFHE) were kept at −20°C until used. The yield of CLHE and CFHE was, respectively, 0.8% and 2% of the dry weight.

### 2.3. High-Performance Liquid Chromatography (HPLC)-Mass Spectrometry (MS) Analysis

HPLC-MS analysis of the hydroethanolic extract of navel orange peels was performed in the Central Laboratory of the Faculty of Postgraduate Studies for Advanced Sciences, Beni-Suef University, Egypt, using the HPLC-MS system, Infinity 1260, Agilent Technologies, Germany, coupled with a diode array detector (DAD). The preparations and analysis were carried out according to the method described by López-Salas et al. [22].

### 2.4. Experimental Animals

Healthy adult male Wistar rats, having a body weight (BW) of 120–140 g and aged 7–9 weeks, were purchased from Helwan Station of Experimental Animals, Egyptian Holding Company for Biological Products and Vaccines (Helwan, Cairo, Egypt). Fourteen days before the experiment onset, all animals were kept under observation for adaptation and to eliminate any inter changeable diseases. Animals were inhabited in polypropylene animal research cages (3 rats/cage) with well-aerated covers under standard temperature (22°C ± 2°C), humidity (55% ± 5%), and 12-hour alternating light and dark cycles. The animals were provided access to tap water and a standard pelleted diet *ad-libitum*. The ethical standards have been adhered to according to the guidelines for IACUC (Institutional Animal Care and Use Committee), Beni-Suef University, Egypt (Ethical Approval Number: BSU/FS/2015/2). Every effort was made to lessen the number of animals and their suffering.

### 2.5. Induction of T2DM

The T2DM model was induced in rats, which were fasting for 16 hours, by intraperitoneal (IP) injection of 60 mg NA/kg BW dissolved in 0.9% NaCl solution. This was followed 15 minutes later by injecting STZ IP at a dose level of 60 mg/kg BW in citrate buffer at pH 4.5 [2, 20]. Ten days postSTZ injections, rats' blood glucose concentrations were screened. Animals that fasted overnight (10–12 hours) were supplied with glucose 3 g/kg BW by oral gavage; then, 2 hours after glucose administration, blood samples were taken from the lateral tail vein, and blood glucose concentrations were measured by a glucometer obtained from Life Scan Corporation, Canada [23]. Rats having blood glucose concentrations >200 mg/dL were assigned as diabetic and selected for further biochemical, physiological, histological, and ultrastructural studies.

### 2.6. Experimental Design

**Scheme 1:**
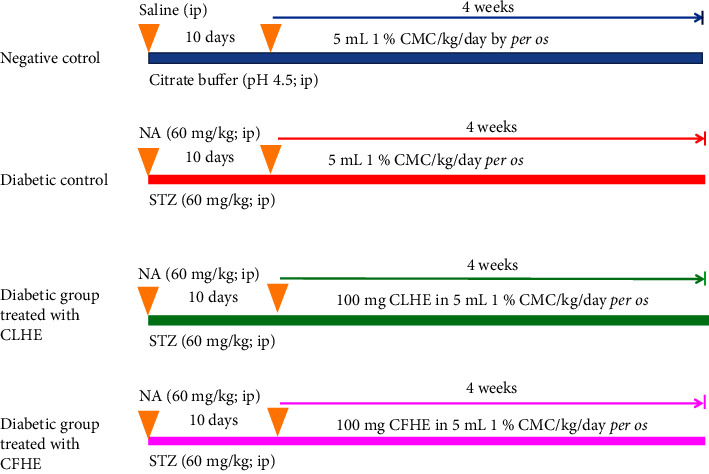
Experimental design and animal grouping. CMC: carboxymethylcellulose, NA: nicotinamide: NA, STZ: streptozotocin, CLHF: *C. scolymus-*leaf hydroethanolic extract, and CFHE: *C. scolymus* flower hydroethanolic extract.

After the acclimation period and induction of T2DM, rats were divided at random into four groups (*n* = 6) (Scheme 1).

#### 2.6.1. Group 1 (Negative Control)

This group comprised healthy rats which received the equivalent volume of 1% carboxymethylcellulose (CMC) as a vehicle every day *per os* for 28 days.

#### 2.6.2. Group 2 (Diabetic Control)

It comprised of rats with diabetes which received an equivalent volume of 1% CMC (as a vehicle) daily *per os* for 28 days.

#### 2.6.3. Group 3 (Diabetic Rats Treated with CLHE)

It included diabetic rats that were treated with CLHE at a dose level of 100 mg/kg BW [20, 24, 25] dissolved in 1% CMC, *per os* daily for 28 days.

#### 2.6.4. Group 4 (Diabetic Rats Treated with CFHE)

It was composed of diabetic rats that were treated with CFHE at a dose level of 100 mg/kg BW [20, 24, 25] dissolved in 1% CMC, *per os* daily for 28 days.

### 2.7. Biochemical Studies

The serum concentration of fructosamine (FA) was estimated based on the procedure of Baker et al. [26]. Hepatic glycogen content was assayed according to Seifter et al. [27]. Based on the techniques developed by Begum et al. [28] and Stalman and Hers [29], the activities of the liver enzymes glucose-6-phosphatase (G6Pase) and glycogen phosphorylase (GP) were measured.

Serum AST and ALT activities were measured according to Schumann and Klauke [30] using reagent kits purchased from HUMAN Gesellschaft für Biochemica und Diagnostica mbH, Magdeburg, Germany.

Serum levels of FFAs were determined according to the method of Duncombe [31]. Serum levels of total cholesterol (TC) [32], HDL-C [32], TAGs [33], LDL-C [34], and VLDL-cholesterol (VLDL-C) [35] were estimated by reagent kits, which were purchased from Spinreact, S.A./S.A.U Ctra.Santa Coloma, 7 E-17176 Sant Esteve De Bas (Gi), Spain.

LPO (lipid peroxidation) [36], SOD (superoxide dismutase) [37], GSH (reduced glutathione) [38], and GPx (glutathione peroxidase) [39] in the liver were detected using JENWAY 6300 spectrophotometer.

### 2.8. Histological Investigation

After dissection, livers were rapidly removed and fixed in 10% neutral phosphate-buffered formalin for 24 hours. Following a thorough rinse in tap water, the samples were dehydrated using a series of ethyl alcohol dilutions (50%, 70%, 90%, 95%, and 100%) in a furnace set at 56°C for 24 hours and then the samples were cleaned with xylene before being submerged into paraffin wax. Sections of 5-*μ*m thickness were made from paraffin wax tissue blocks with a sliding microtome. For a standard examination, the tissue sections were mounted on glass slides, dewaxed, and stained with hematoxylin and eosin (H&E) [40]. The examination was carried out using a light electric microscope.

### 2.9. Ultrastructural Preparations

The liver specimens on day 30 were cut into small specimens, about one mm^3^, and were then instantly fixed for 18 to 24 hours at 4°C in fresh 3% glutaraldehyde-formaldehyde. Thereafter, the specimens were processed for preparation and staining of semithin sections and preparation of ultrathin sections that were investigated using a Joel CX 100 transmission electron microscope according to Bozzola and Russell [41] and Ahmed et al. [20].

### 2.10. Immunohistochemistry of Liver

Immunolocalization for hepatic tumor necrosis factor-*α* (TNF-*α*) was applied on sections of 5-*μ*m thickness and stained with the streptavidin-biotin-peroxidase staining technique [20, 42, 43] using an antisera containing 1^ry^ antibodies for rat anti-TNF-*α* (Santa Cruz, CA, USA), biotinylated 2^ry^ antibodies and streptavidin horseradish peroxidase (Dako-K0690; Dako Universal LSAB Kit), and diaminobenzidine tetrahydrochloride (DAB) substrate kit (Sigma-D5905; Sigma–Aldrich Company Ltd., Gillingham, UK) for 10 min for immunolabelling. All prepared sections were treated under the same conditions with the same antibody concentrations simultaneously, so the immunostaining among the different groups was comparable.

### 2.11. Statistical Analysis

The mean and standard deviation (SD) were used to express the results. To compare the tested groups, a one-way analysis of variance (ANOVA) with PC-STAT (version 1A (C) copyright 1985, University of Georgia, Georgia, USA) was used, followed by the least significant difference (LSD) test [44]. Values of *p* < 0.05 were statistically significant while values of *p* ≥ 0.05 were not.

## 3. Results

### 3.1. HPLC-MS Analysis of CLHE and CFHE

**Figure 1:**
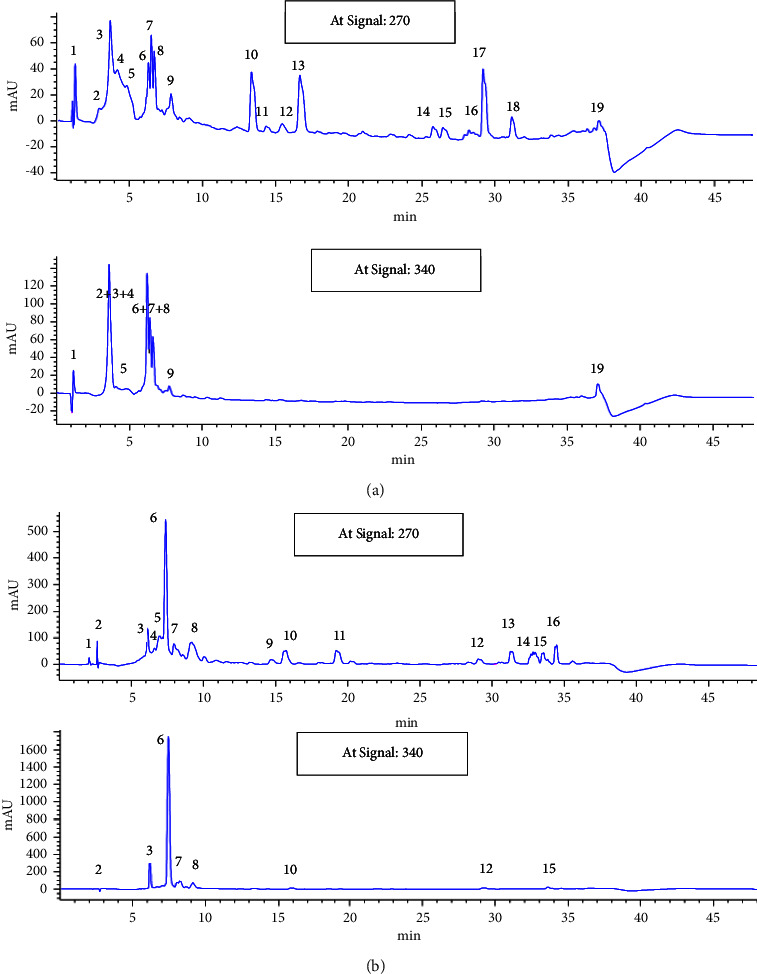
(a) HPLC-MS analysis of CLHE indicating the presence of phenolic acids (gallic acid (1), quinic acid (2), chlorogenic acid (3), rosmarinic acid (4), and caffeic acid (5)), cynarine isomers (6, 7, 8, and 9), flavonoids especially flavones luteolin and apigenin (10, 11, 12, and 13), cynarasaponin isomers (14, 15, 16, and 17), hydroxyoctadecadienoic acid (18), and linolenic acid (19). (b) HPLC-MS analysis of CFHE indicating the presence of phenolic acids and cynarine isomers (1–8), flavonoids especially flavones luteolin and apigenin (9, 10, and 11), cynarasaponin isomer (12), and polyunsaturated fatty acids (13–16).

HPLC-MS analysis of CLHE showed the presence of several categories of compounds including phenolic acids (gallic acid, quinic acid, chlorogenic acid, rosmarinic acid, and caffeic acid), cynarine isomers, flavonoids (especially flavones luteolin and apigenin), cynarasaponin isomers, hydroxyoctadecadienoic acid, and linolenic acid (Figure 1(a)).

Similarly, HPLC-MS analysis of CFHE indicated the presence of phenolic acids, cynarine isomers, flavonoids especially flavones luteolin and apigenin, cynarasaponin isomer, polyunsaturated fatty acids, hydroxyoctadecadienoic acid, and linolenic acid.

### 3.2. Biochemical Effects

The serum FA level and hepatic GP and G6Pase activities were remarkably (*p* < 0.05) elevated, while hepatic glycogen content was statistically markedly (*p* < 0.05) depleted in the diabetic control in comparison with the negative control rats. CLHE and CFHE therapy of the diabetic rats resulted in a discernible (*p* < 0.05) improvement in the serum FA level, liver GP, and the G6Pase activities and glycogen content in comparison with the diabetic control. Treatments with CLHE appeared to have a stronger impact on improving the serum FA level, liver glycogen content, and the G6Pase enzyme activity (Table 1).

Serum AST and ALT activities were markedly (*p* < 0.05) raised in the diabetic control rats in relation to the negative control rats. At the same time, they were remarkably decreased in the diabetic group supplemented with CLHE and CFHE in relation to the diabetic control group (Table 2). While the effect of CLHE on ALT activity was statistically significant (*p* < 0.05), but the effect on AST activity was not (*p* ≥ 0.05).

Regarding serum lipid profile (Table 3), the rats with diabetes exhibited significant elevations (*p* < 0.05) in TC, LDL-C, VLDL-C, and FFAs levels and significant reduction (*p* < 0.05) in HDL-C levels compared to negative control ones. The treatment with CLHE and CFHE produced a remarkable amelioration of all these lipid profile variables to various extents; and CFHE appeared to be more effective in decreasing the elevated serum lipids.

Table 4 shows the effects of CLHE and CFHE on oxidative stress and antioxidant defence system biomarkers in the liver of the study groups. Liver LPO was remarkably (*p* < 0.05) upregulated in the diabetic control, while it was decreased (*p* < 0.05) in diabetic rats supplied with CLHE and CFHE in relation to the diabetic control. The level of the nonenzymatic antioxidant (GSH) and activity of the antioxidant enzymes including SOD and GPx decreased considerably (*p* < 0.05) in the liver of the diabetic control. The supplementation of the diabetic rats with CLHE and CFHE resulted in a detectable increase in the liver GSH content and SOD and GPx activities to various extents. Despite the effect of both CLHE and CFHE on GPx and SOD activities were statistically significant, the impact on GSH content was not (*p* ≥ 0.05). CLHE seemed to be more potent than CFHE in reducing the elevations in the liver LPO.

**Figure 2:**
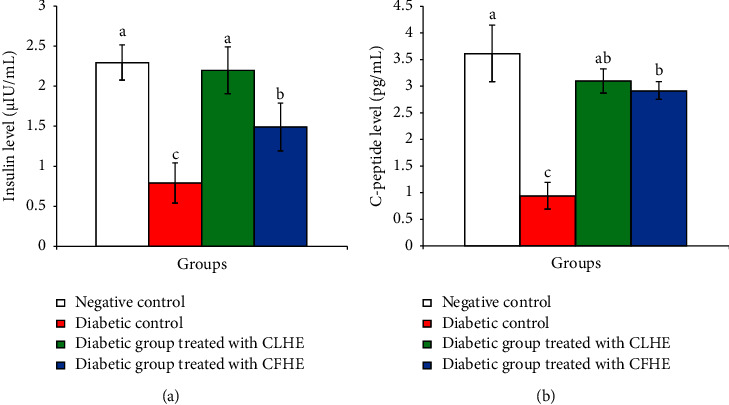
Effect of CLHE and CFHE on serum insulin (a) and C-peptide (b) levels among the study groups. Data are expressed as mean ± SD. For each variable, values that do not share the same superscript letter (a, b, and c) were significantly different at *p* < 0.05. CLHF: *C. scolymus* leaf hydroethanolic extract and CFHE: *C. scolymus* flower hydroethanolic extract.

The serum insulin and C-peptide levels were statistically significantly depleted in diabetic control rats compared with the negative control ones. The treatment with CLHE and CFHE induced a statistically significant increase in the lowered levels, and CLHE was the most potent (Figure 2).

### 3.3. Histological Changes

**Figure 3:**
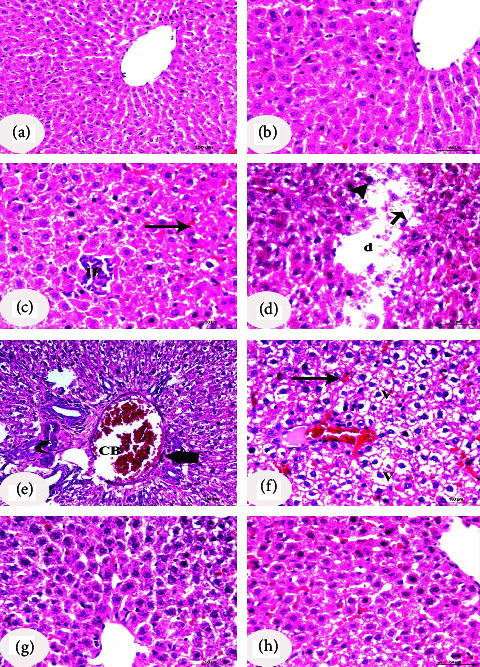
Photomicrographs of liver sections of rats of the 4 study groups. (a, b) Photomicrographs of the negative control group showed hepatocytes arranged in an orderly and normal hepatic architecture with normal hepatocyte morphology and organized hepatic cell cords radiating from the central vein. (b) is a higher magnification of (a). (c-f) Photomicrographs of the diabetic control group showed disordered hepatocyte, dissolution of cytoplasm (d), monocellular leukocytic infiltration (IF), hydropic degeneration and vacuolations (v), karyomegaly of the nucleus or hyperchromatic nucleus (arrowhead), karyolysis of the nucleus (short arrow), and dilated congested portal vein (CB). The proliferation of bile ducts and degenerative changes in the wall of some bile ducts are also observed (curved arrow). Dilated hyperemic sinusoids (long arrow) and thickened wall (thick arrow) are also seen. Diabetic groups treated with CLHE (g) and CFHE (h) showed amelioration of hepatocytes' microscopical structure, sinusoid, and central vein. Scale bars of photomicrographs (a), (e), and (f) = 100 *μ*m and scale bars of photomicrographs (b), (c), (d), (g), and (h) = 50 *μ*m.

H&E staining results obtained upon histological examination are shown in Figure 3. The hepatocytes in the negative control group were distributed in an ordered fashion and displayed a typical hepatic architecture, including organized hepatocytic cords, normal hepatocyte morphology, and a portal vein with sinusoidal cords (Figures 3(a) and 3(b)). On the other hand, the most significant alterations in the diabetic control rats' livers were disorderly hepatocyte, cytoplasm dissolution, monocellular leukocytic infiltration, karyomegaly, hyperchromatic nuclei, nucleus karyolysis, and dilated congested portal vein. The proliferation of bile ducts and degenerative changes in the wall of some bile ducts were also observed. Dilated hyperemic sinusoids and thickened walls were also seen (Figures 3(c)–3(f)). Treated groups with CLHE and CFHE showed amelioration of hepatocytes, sinusoid, and central vein (Figures 3(g) and 3(h)).

### 3.4. Effect on TNF-*α* Expression by Immunohistochemical Investigation

**Figure 4:**
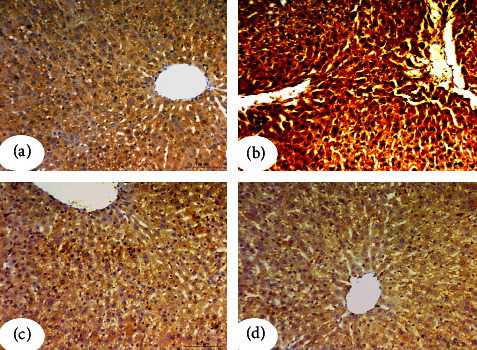
Photomicrographs from immunohistochemistry assays for liver TNF-*α* in the 4 study groups. (a) Photomicrograph of the liver of the negative control group. (b) Photomicrograph of the liver of the diabetic control group showed a marked increase in the intensity of TNF-*α* in the cytoplasm of the hepatocyte. Diabetic rats of pancreas islets treated with CLHE (c) and CFHE, (d) respectively, showed the reduced intensity of TNF-*α*. Scale bars of figures a and b = 100 *μ*m and scale bars of figures c and d = 50 *μ*m.

Immunohistochemistry sections of the negative control group showed no reaction for TNF-*α* (Figure 4(a)), while the diabetic control group revealed a marked increase in the intensity of TNF-*α* in the cytoplasm of hepatocytes (Figure 4(b)). Treatment with CLHE and CFHE showed a noticeable reduction in the TNF-*α* in hepatocyte intensity (Figures 4(c) and 4(d)).

### 3.5. Ultrastructural Effects

**Figure 5:**
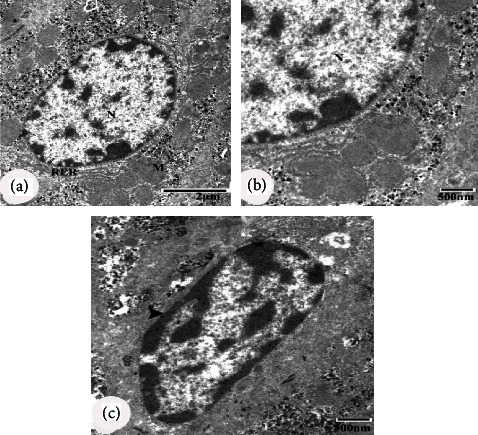
Electron microscopic (EM) micrographs of hepatocytes of the negative control group. (a, b) EM micrographs showing normal hepatocytes, nucleus (N), mitochondria (m) and rough endoplasmic reticulum (RER) with their preserved structures. (c) EM micrograph showing Kupffer cell (arrowhead). Scale bars of figure a = 2 *μ*m, and scale bars of figures b and c = 500 nm.

**Figure 6:**
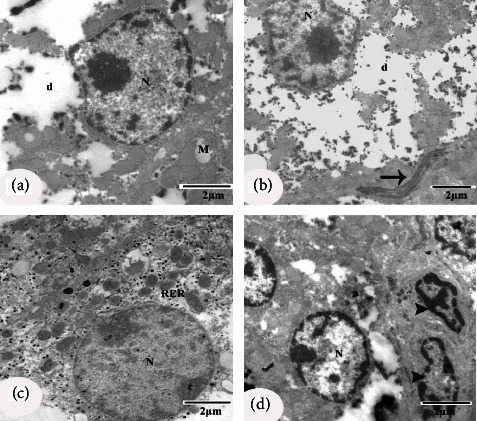
EM micrographs of hepatocytes of the diabetic control rats. (a–d) EM micrographs showing the dissolution of cytoplasm (d), nucleus (N) with irregular nuclear membrane, collagen fibre bundle (arrow), damaged mitochondria with ill-defined cristae (m), and fragmentation of rough endoplasmic reticulum (RER). (d) EM micrograph showing proliferated Kupffer cell, where nucleus appeared with heterochromatin clumps adjacent to the irregular nuclear membrane (arrowhead). Scale bars of figures a, b, c, and d = 2 *μ*m.

**Figure 7:**
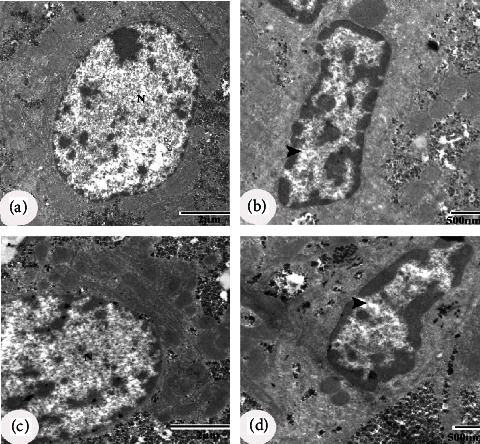
EM micrographs of hepatocytes of the diabetic rats treated with CLHE (a, b) showing intact nucleus (N) and regular Kupffer cell (arrowhead) and diabetic rats treated with CFHE (c & d) showing normal nucleus (N) and Kupffer cell (arrow head) with their preserved structures. Scale bars of figures a and c = 2 *μ*m, and scale bars of figures b and d = 500 nm.

Electron microscopic examination of the negative control group revealed normal-appearing hepatocytes with a normal structure of the nucleus, mitochondria, and rough endoplasmic reticulum (RER) (Figures 5(a) and 5(b)). Kupffer cells are shown in Figure 5(c). Hepatocytes of diabetic control rats revealed cytoplasm dissolution, a nucleus with irregular nuclear membrane, dilated intercellular space containing collagen fibre bundle, damaged mitochondria with ill-defined cristae, and fragmentation of RER (Figures 6(a)–6(c)). The nucleus of the Kupffer cells appeared with heterochromatin clumps adjacent to the irregular nuclear membrane (Figure 6(d)). Hepatocytes of diabetic rats treated with CLHE showed an intact nucleus and regular Kupffer cells (Figures 7(a) and 7(b)). Diabetic rats treated with CFHE showed normal nuclei with their Kupffer cells preserved (Figures 7(c) and 7(d)).

## 4. Discussion

DM includes a group of chronic metabolic derangements featured by hyperglycemia due to absolute or relative insulin deficiency and/or IR that results from insulin receptor or postreceptor defects. It affects metabolisms of carbohydrates, proteins, and fats as well as has deteriorative effects on hepatic, renal, and pancreatic islet beta cells [45]. Glucotoxicity, lipotoxicity, and oxidative stress are some of the mechanisms that contribute to the excessive formation of reactive oxygen species and reactive nitrogen species in this situation [2].

In the presented study, a significant rise in the serum FA levels in rats with DM was recorded. The serum level of FA is a good and accurate biomarker of the glycemic state since it measures the average blood glucose level over 2-3 weeks. The supplementation of diabetic rats with CLHE and CFHE showed a significant improvement in the FA level which is consistent with the results of Alves et al. [46]. The improvement in serum fructosamine levels, as a result of the treatment of diabetic rats with CLHE and CFHE, was associated with the increase in serum insulin and C-peptide levels. Thus, the improvement in the glycemic state by treatment may be attributed to the insulinogenic and insulinotropic effects of CLHE and CFHE. This attribution was supported by previous publications [47, 48]. The protective actions of artichoke extract on the pancreatic *β*-cells and enhancement of their regeneration, in addition to its stimulatory effects on insulin release from cells that survived damage by the diabetes-inducing agent, may cause of the rise in serum insulin and C-peptide levels [48].

**Scheme 2:**
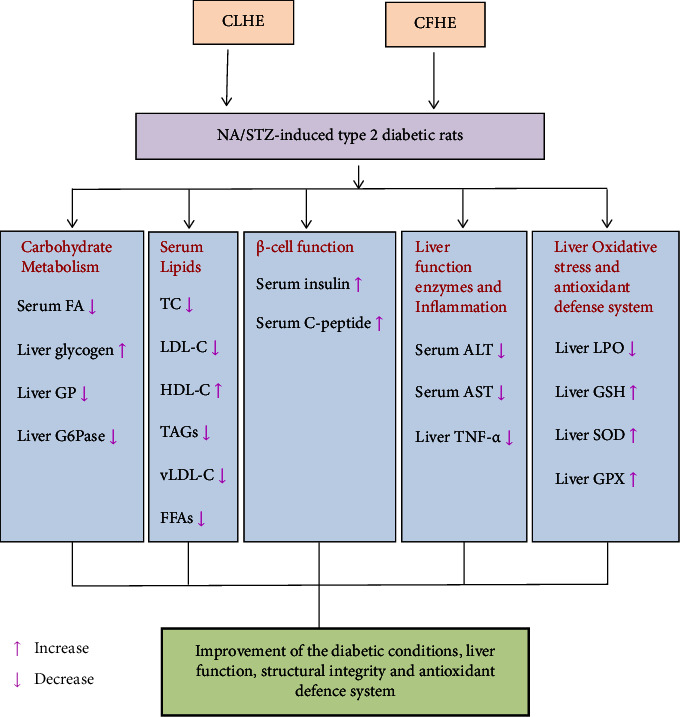
Schematic illustrative figure to depict the suggested hypothesis of the modes of actions of CLHE and CFHL in NA/STZ-induced diabetic rats.

Our results showed a significant drop in the liver glycogen content and a profound elevation of the liver GP and G6Pase activities in the diabetic control rats. These results agreed with Ahmed et al.[49] and Ahmed [50], who reported a marked depletion of the liver glycogen content and elevation of the GP and G6Pase activities in NA/STZ-induced type 2 diabetic rats. The increase in GP activity associated with glycogen depletion reflects the increased glycogenolysis, which contributes to the increase in hepatic glucose output in T2DM. Glucose-6-phosphate, which serves as a metabolic hub to link glycolysis, the pentose phosphate route, glycogen synthesis, *de novo* lipogenesis, and the hexosamine pathway, may be depleted as a result of the increase in G6Pase activity observed in type 2 diabetic rats in the present study [51]. The treatments of diabetic rats with CLHE and CFHE revealed a significant increase in the liver glycogen content and significant decreases in the GP and G6Pase activities; and these improvements may, in turn, lead to a decrease in the hepatic glucose production and may attribute to the antihyperglycemic and antidiabetic effects of CLHE and CFHE (Scheme 2).

In the present study, serum AST and ALT activities exhibited a significant elevation in the diabetic group when compared with the negative control. These changes agreed with Ahmed [52] and Parmar et al. [53], who indicated a marked increase in the AST and ALT levels in rats with DM. The increase in serum AST and ALT activities in diabetic rats may be attributed to their greater need for gluconeogenic substrates and their leakage from the damaged hepatocytes [2, 52]. The treatments of NA/STZ-induced diabetic rats with CLHE and CFHE resulted in a marked decrease in these two cytoplasmic enzymes in serum, which may be secondary to the improvements in the diabetic conditions and liver function and structural integrity. These results go parallel with El-Deberky et al. [25] and Heidarian and Rafieian-Kopaei [54], who revealed that *Cynara scolymus* extracts produced a significant decrease in the elevated AST and ALT activities in association with amelioration of the liver's histological architecture in thioacetamide-treated rats and in lead-intoxicated rats, respectively.

The present study showed an elevation in serum FFAs levels in the diabetic control rats. These results are in concurrence with the data of Ahmed [50]. The persistent elevations in circulating FFAs levels have an important role in developing IR and *β*-cell dysfunction in T2DM [2, 55]. Supplementation of diabetic rats with CLHE and CFHE leads to a partial decrease in serum FFAs levels compared to the diabetic control. This improvement in FFAs levels may have an important role in the decrease of insulin resistance produced by treatments with CLHE and CFHE as indicated in our previous publication [20].

In NA/STZ-induced diabetic rats, serum levels of TC, TAGs, LDL-C, and VLDL-C increased significantly, whereas there was a reduction in the HDL-C levels compared to the negative control rats. Also, a significant reduction in TC, TAGs, LDL-C, and VLDL-C levels in diabetic rats was attained by treatment with CLHE and CFHE, which is more potent. These changes are consistent with Soofiniya and Heidarian [56], who found that the oral supplementation of *C. scolymus* leaf aqueous extract for 21 days significantly reduced TC, TAGs, LDL-C, VLDL-C, and glucose levels in the serum of STZ-induced diabetic rats. It was reported that *C. scolymus* has hypocholesterolemic efficacy by reducing the cholesterol synthesis [57]. This action may be attributed to the finding that artichokes contain luteolin and cynarine, which play an important role in suppressing the cholesterol and TAGs synthesis, and glucoside release. It was found that luteolin by beta-glucosidase in the digestive tract may result in a suppression of up to 60% of cholesterol biosynthesis [58]. However, a remarkable diminishment of plasma LDL-C, VLDL-C, and an elevation of HDL-C agree with Cieslik et al. [59] who revealed a declining tendency in TC, LDL-C, and VLDL-C when diets were supplied with powder of Jerusalem artichoke. Meanwhile, Taylor [60] showed a 10–15% reduction in TC/LDL-C and LDL-C/HDL-C ratios probably due to the fact that leaf contains active constituents such as flavonoids and caffeoylquinic acid, which possess antihyperlipidemic effects. These compounds belong to polyphenols, chlorogenic acid, cynarine, luteolin scolymosides, and cynarosides [61]. Thus, based on the obtained results, it can be concluded that CLHE and CFHE have antidiabetic action by possessing glucose and cholesterol-lowering effects (Scheme 2).

Our results showed significant downregulation in the SOD and GSH content in diabetic control rats' hepatic tissue compared to the negative control rats, which agreed with Dewanjee et al. [62]. The decreased SOD levels in diabetic rats were almost reverted to normal status after the extract treatment. The downregulation of the liver GSH level leads to augmented oxidative stress [63]. Repression in SOD activity in diabetic rats could result from inactivation by H_2_O_2_ or glycosylation of the enzyme in DM [64]. In parallel with our study, Colak et al. [65] and Ahmadi et al. [66] found that the treatment with CLHE improved the lowered liver SOD activity, respectively, in CCl_4_- and diazinon-induced liver injuries in rats.

Diabetic control rats showed an important reduction in GPx in hepatic tissue. These deteriorations in GPx activity were reduced after treatment with CLHE and CFHE. These results agreed with Kaymaz et al. [67], who reported a significant increase in the hepatic GPx activity by treatment with artichoke leaf extract in alpha-amanitin-induced hepatotoxicated rats. LPO activity increased in the liver homogenates in the diabetic control group compared to the negative control but decreased in the diabetic groups treated with CLHE and CFHE compared to the diabetic control. In agreement, Matkovics et al. [68] and Anwar and Meki, [69] found that in the liver of diabetic rats, LPO increased. Colak et al. [65] and Ahmadi et al. [66] revealed that the treatment with CLHE significantly reduced the liver LPO elevations, respectively, in CCl_4_- and diazinon-induced liver injuries in rats.

The present study on hepatocytes from diabetic control rats showed a significant increase in the expression of the proinflammatory cytokine, TNF-*α*, in the liver. Treatment with CLHE and CFHE showed a significant reduction in the liver TNF-*α* expression consistent with the absence of inflammatory cells' infiltration in histological sections and reflects the anti-inflammatory effects of these extracts. Ahmadi et al. [66] agreed with our results of a marked decrease in hepatic TNF-*α* expression in the liver of diazinon-induced liver injury in rats treated with CLHE.

Histological changes may be due to the hepatotoxic effect of STZ. Diabetic livers showed disorderly hepatocytes, cytoplasm dissolution, mononuclear leukocytic infiltration, a proliferation of bile ducts, and degenerative changes in the wall of some bile ducts. After treatment with CLHE and CFHE, marked amelioration was observed. These changes are similar to the results of Arya et al. [70].

The present EM ultrastructure results of the NA/STZ-diabetic liver showed cytoplasm dissolution, irregular nuclear membrane, collagen fibre bundle, damaged mitochondria with ill-defined cristae, and fragmentation of RER. These results are in concurrence with Lucchesi et al. [71], who revealed similar liver ultrastructural changes in alloxan-induced diabetic rats. Moreover, Balazs and Halmos [72] showed that dilated intercellular space contains a collagen fibre bundle. Can et al. [73] observed dense degenerative mitochondria and a RER in the diabetic group.

In our opinion, the improvement effects of CLHE and CFHE on liver function and histological and ultrastructural deteriorations in diabetic rats may be attributed to their antioxidant and anti-inflammatory properties (Scheme 2). In support to this attribution, Ahmadi et al. [66] stated that the ameliorative effects of CLHE against diazinon-induced liver injury are due to its antioxidant and anti-inflammatory properties.

The antidiabetic effects and the preventive effects of CLHE and CFHE against liver injury in NA-STZ-induced diabetic rats may be attributed to their constituents which have several biological activities. These constituents include phenolic acids, cynarine isomers, flavones (luteolin and apigenin), cynarasaponin isomers, and polyunsaturated fatty acids.

## 5. Conclusions

In conclusion, the present study provides evidence that both CLHE and CFHE have antidiabetic effects. However, further clinical studies are required to assess the efficacy and safety of CLHE and CFHE in type 2 diabetic human beings. Both CLHE and CFHC have ameliorating effects on the liver function and histological and ultrastructural integrity that may be mediated *via* the suppression of oxidative stress and inflammation as well as enhancement of the antioxidant defence system. To pinpoint the molecular mechanisms of CLHE and CFHE, more research studies are required to examine the impact on mediators of insulin resistance and inflammation.

## Figures and Tables

**Table 1:** The effect of CLHE and CFHE on the serum FA level, liver glycogen content, and the liver GP and G6Pase activities among the study groups.

Groups (*n* = 6)	Serum FA (*μ*mole/L)	Liver glycogen (mg/g tissue)	GP GP (mg *P*_*i*_ liberated/g tissue/hr)	G6Pase (mg *P*_*i*_ liberated/g tissue/hr)
Negative control	179.45 ± 27.44^c^	51.08 ± 11.00^a^	14.87 ± 2.30^c^	3.21^c^ ± 38.20
Diabetic control	958.52 ± 84.53^a^	9.20 ± 1.94^c^	37.68 ± 2.79^a^	56.68 ± 6.20^a^
Diabetic treated with CLHE	228.15 ± 8.97^c^	43.18 ± 10.24^ab^	21.15 ± 2.01^b^	1.98^b^ ± 49.3
Diabetic treated with CFHE	409.03 ± 22.05^b^	39.68 ± 6.05^b^	20.50 ± 1.10^b^	45.26 ± 2.94^b^

Data were expressed as mean ± SD. For each variable, values that do not share the same superscript letter (a, b, and c) were significantly different at *p* < 0.05. FA, fructosamine; GP, glycogen phosphorylase; G6Pase, glucose-6-phosphatase; CLHF, *C. scolymus* leaf hydroethanolic extract; CFHE, *C. scolymus* flower hydroethanolic extract.

**Table 2:** The effect of CLHE and CFHE on ALT and AST activities among the study groups.

Groups (*n* = 6)	ALT (U/L)	AST (U/L)
Negative control	23.97 ± 4.41^c^	93.33 ± 10.95^c^
Diabetic control	52.86 ± 21.22^a^	129.73 ± 14.82^a^
Diabetic treated with CLHE	39.33 ± 16.15^b^	126.86 ± 13.65^a^
Diabetic treated with CFHE	43.71 ± 15.70^b^	109.43 ± 33.19^b^

Data were expressed as mean ± SD. For each variable, values that do not share the same superscript letter (a, b, and c) were significantly different at *p* < 0.05. ALT, alanine transaminase; AST, aspartate transaminase; CLHF, *C. scolymus* leaf hydroethanolic extract; CFHE, *C. scolymus* flower hydroethanolic extract.

**Table 3:** Effect of CLHE and CFHE on TC, LDL-C, HDL-C, TAGs, VLDL-C, and FFAs levels among the study groups.

Group (*n* = 6)	TC (mg/dL)	LDL-C (mg/dL)	HDL-C (mg/dL)	TAGs (mg/dL)	VLDL-C (mg/dL)	FFAs (*n*mole/L)
Negative control	50.50 ± 2.08^d^	18.55 ± 1.62^d^	27.5 ± 1.20^a^	26.18 ± 2.25^d^	5.24 ± 0.44^d^	16.10 ± 0.37^b^
Diabetic control	114.33 ± 8.33^a^	84.43 ± 14.50^a^	22.52 ± 1.57^c^	45.93 ± 2.21^a^	9.20 ± 0.45^a^	44.43 ± 6.07^a^
Diabetic treated with CLHE	92.66 ± 11.52^b^	59.65 ± 13.69^b^	25.03 ± 1.62^b^	35.1 ± 3.70^b^	6.91 ± 0.71^b^	42.88 ± 2.99^a^
Diabetic treated with CFHE	62.00 ± 5.41^c^	32.4 ± 6.49^c^	23.53 ± 1.64^c^	30.33 ± 1.20^c^	6.1 ± 0.27^c^	40.10 ± 3.19^a^

Data were expressed as mean ± SD. For each variable, values that do not share the same superscript letter (a, b, c, and d) were significantly different at *p* < 0.05. TC, total cholesterol; LDL-C, low-density lipoprotein cholesterol; HDL-C, high-density lipoprotein cholesterol; TAGs, triglycerides; VLDL-C, very-low-density lipoprotein-cholesterol; FFAs, free fatty acids; CLHF, *C. scolymus* leaf hydroethanolic extract; CFHE, *C. scolymus* flower hydroethanolic extract.

**Table 4:** Effect of CLHE and CFHE on liver LPO, GSH content, and SOD and GPx activities among the study groups.

Group (*n* = 6)	LPO (*n*mole/100 mg tissue (hr))	GSH (*n*mole ∕100 mg tissue)	SOD (U∕gm tissue)	GPx (mU∕100 mg tissue)
Negative control	24.85 ± 2.94^b^	58.38 ± 5.93^a^	18.66 ± 0.51^a^	117.66 ± 13.35^a^
Diabetic control	59.80 ± 1.79^a^	22.71 ± 0.34^b^	16.50 ± 0.54^c^	66.66 ± 16.64^c^
Diabetic group treated with CLHE	28.25 ± 1.59^c^	23.76 ± 3.85^b^	17.33 ± 0.52^b^	92.33 ± 3.65^b^
Diabetic group treated with CFHE	42.00 ± 4.17^d^	22.96 ± 0.37^b^	17.50 ± 0.83^b^	97.33 ± 31.08^ab^

Data were expressed as mean ± SD. For each variable, values that do not share the same superscript letter (a, b, and c) were significantly different at *p* < 0.05. LPO, lipid peroxidation; GSH, glutathione; SOD, superoxide dismutase; GPx, glutathione peroxidase; CLHF, *C. scolymus* leaf hydroethanolic extract; CFHE, *C. scolymus* flower hydroethanolic extract.

## Data Availability

The data supporting the current study are included in the article.

## Abbreviations

ALT: Alanine transaminase
AST: Aspartate transaminase
*C scolymus*: *Cynara scolymus*
CFHE: *C. scolymus* flower hydroethanolic extract
CLHE: *C scolymus* leaf hydroethanolic extract
CMC: Carboxymethylcellulose
DAB: Diaminobenzidine
DAGs: Diacylglycerols
DM: Diabetes mellitus
EM: Electron microscopic
ER: Endoplasmic reticulum
FFAs: Free fatty acids
GPx: Glutathione peroxidase
GSH: Reduced glutathione
G6Pase: Glucose-6-phosphatase
GP: Glycogen phosphorylase
H&E: Hematoxylin and eosin
HDL-C: High-density lipoprotein-cholesterol
IACUC: Institutional animal care and use committee
IR: Insulin resistance
IF: Inflammation
IP: Intraperitoneal
LDL-C: Low-density lipoprotein-cholesterol
LPO: Lipid peroxidation
NA: Nicotinamide
RER: Rough endoplasmic reticulum
SD: Standard deviation
SOD: Superoxide dismutase
STZ: Streptozotocin
T2DM: Type 2 diabetes mellitus
TC: Total cholesterol
TAGs: Triacylglycerols
TNF-*α*: Tumor necrosis factor-*α*.
